# Phylogeny of genera in Maleae (Rosaceae) based on chloroplast genome analysis

**DOI:** 10.3389/fpls.2024.1367645

**Published:** 2024-03-26

**Authors:** Jiahui Sun, Dan Zhao, Ping Qiao, Yiheng Wang, Ping Wu, Keren Wang, Lanping Guo, Luqi Huang, Shiliang Zhou

**Affiliations:** ^1^ State Key Laboratory for Quality Ensurance and Sustainable Use of Dao-di Herbs, National Resource Center for Chinese Materia Medica, China Academy of Chinese Medical Sciences, Beijing, China; ^2^ Dexing Research and Training Center of Chinese Medical Sciences, China Academy of Chinese Medical Sciences, Dexing, China; ^3^ College of Pharmacy, Guizhou University of Traditional Chinese Medicine, Guiyang, China; ^4^ State Key Laboratory of Systematic and Evolutionary Botany, Institute of Botany, Chinese Academy of Sciences, Beijing, China; ^5^ Institute of Highland Forest Science, Chinese Academy of Forestry, Kunming, China

**Keywords:** chloroplast genome, generic classification, Maleae, phylogeny, Rosaceae

## Abstract

In Rosaceae, the replacement of the traditional four-subfamily division (Amygdaloideae or Prunoideae, Maloideae, Rosoideae, and Spiraeoideae) by the three-subfamily division (Dryadoideae, Rosoideae, and Amygdaloideae), the circumscription, systematic position, and phylogeny of genera in Maleae need to be reconsidered. The study aimed to circumscribe Maleae, pinpoint its systematic position, and evaluate the status of all generally accepted genera in the tribe using complete chloroplast genome data. Results indicated that Maleae consisted of pome-bearing genera that belonged to Maloideae as well as four genera (*Gillenia*, *Kageneckia*, *Lindleya*, and *Vauquelinia*) that were formerly considered to be outside Maloideae. The tribe could be subdivided into four subtribes: Gilleniinae (*Gillenia*), Lindleyinae (*Kageneckia* and *Lindleya*), Vaugueliniinae (*Vauquelinia*), and Malinae (all other genera; the core Maleae). Among the 36 recognized genera, *Aria*, *Docyniopsis*, *Chamaemespilus*, and *Mespilus* were not considered distinct and more research is needed to determine the taxonomic status of *Rhaphiolepis* from *Eriobotrya*. Within the core Maleae, five groups were revealed, whereas *Sorbus* L. was split as its members belonged to different groups.

## 1 Introduction

Molecular systematics has deeply changed the classification of the Rosaceae family from the subdivision of subfamilies to species. This family includes approximately 2,950 species in 91 genera, of which many are economically important such as apples, pears, peaches, strawberries, roses, and cherries (Christenhusz and Byng, 2016). The conventional four-subfamily division (Amygdaloideae, Maloideae, Rosoideae, and Spiraeoideae) was replaced by a three-subfamily division [Amygdaloideae (including Maloideae and Spiraeoideae), Dryadoideae (divided from Rosoideae), and Rosoideae] that was based on molecular data (Potter et al., 2002, 2007; Xiang et al., 2016; Zhang et al., 2017). In the three-subfamily division, subfamily Maloideae (2n = 34) with inferior ovaries was merged with the subfamily Amygdaloideae (2n = 16, 18) with superior ovaries, whereas the species of Maloideae were grouped into Maleae (Pyrinae; Potter et al., 2007). Besides, the four capsule-producing genera *Gillenia* Moench (= *Porteranthus* Britton, 2n = 18), *Kageneckia* Ruiz & Pav. (2n = 34), *Lindleya* Kunth (2n = 34), and *Vauquelinia* Corrêa ex Bonpl. (2n = 30) that formerly included in Spiraeoideae showed a closer genetic relationships with the pome-bearing Maleae (Evans & Campbell, 2002; Campbell et al., 2007; Potter et al., 2007; Li et al., 2012; Lo & Donoghue, 2012; Xiang et al., 2016; Zhang et al., 2017; Sun et al., 2018). Sun et al. (2018) showed that the four capsule-producing genera and the pome-bearing genera comprise the tribe Maleae and that the latter forms the core Maleae.

Taxonomic classification within the core Maleae has been a subject of considerable debate, primarily due to the complex interplay of polyploidy, hybridization, and apomixis. Robertson et al. (1991) identified 28 genera of pome-bearing species, while Gu & Spongberg (2003) delineated 16, underscoring the variability in genus circumscriptions. The pome-bearing species with 2n = 34 (x = 17) were once believed to be originated by a genome merge between species of Amygdaloideae (x = 8) and Spiraeoideae (x = 9) (Sax, 1932; Stebbins, 1950; Phipps et al., 1991). However, such an inter-subfamilial origin was not supported by morphological data (Phipps et al., 1991). Evans & Campbell (2002) proposed a hypothesis of aneuploidy (x = 17 from x = 18) and *Gillenia* as a possible ancestor based on the nuclear gene GBSSI (granule-bound starch synthase I). Recent studies based on genome information supported aneuploidization events that occurred approximately 50 million years ago (Velasco et al., 2010; Considine et al., 2012). Nonetheless, the presence of two copies of the GBSSI locus in *Amelanchier* Medik (Evans et al., 2000). may indicate that the allotetraploid origin of genera is one of the pathways in the evolution of the core Maleae, which obscures the distinction among genera and inevitably complicates taxonomic studies. Intergeneric hybridization is rather common in the core Maleae (Robertson et al., 1991, Figure 1), owing to the nature of multiple copies of genes in polyploids and apomixis for the survival of hybrids as well as the close genetic relationships among the genera.

The pome-bearing core Maleae were subdivided into one group that included the connate endocarps and another that included the polypyrenous drupes (Hutchinson, 1964; Schulze-Menz and Melchior, 1964; Phipps et al., 1990; Kalkman and Kubitzki, 2004). In fact, the subdivision of the core Maleae beyond the generic level based on morphological features is considered unreliable due to their complexity (Phipps et al., 1991).

Previous the attempts for the circumscription of genera were of diverse opinions due to their understanding of morphological variations. In any case, there were 36 genera have been recognized previously (Phipps et al., 1990; Robertson et al., 1991; Sun et al., 2018, **Supplementary Table S1**). *Crataegus* L., *Eriobotrya* Lindl., *Mespilus* L., and *Rhaphiolepis* Lindl. were taxonomically uncontroversial; however, the genetic divergence of each *Mespilus* from *Crataegus* and *Rhaphiolepis* from *Eriobotrya* were too small for generic ranking based on molecular evidence (Lo and Donoghue, 2012; Sun et al., 2018). Morphological data did not help to effectively address any taxonomic problems based on phylogeny, especially in the *Sorbus*-related, *Malus*-related, and *Photinia*-related genera. Therefore, molecular tools might help to reconstruct the phylogeny of Maleae (Xiang et al., 2016; Zhang et al., 2017; Sun et al., 2018). Xiang et al. (2016) built a phylogeny of Rosaceae with sequences of 113 genes in which 16 genera of the core Maleae were sampled. Zhang et al. (2017) sampled 25 genera of the core Maleae in their study of Rosaceae. Sun et al. (2018) used 15 chloroplast regions to reconstruct the phylogeny of Maleae and concluded that “it is still premature to make a formal taxonomic treatment for these genera” in the tribe.

In this study, we used the complete chloroplast genome of 35 genera of Maleae except *Chamaemeles* Lindl. to reconstruct a well-presented phylogeny of the tribe Maleae. Based on our chloroplast genome data and molecular information provided by previously published studies (Xiang et al., 2016; Zhang et al., 2017; Sun et al., 2018), a validated taxonomy of the genera in Maleae will be presented after considering all the morphological differences and genetic divergences.

## 2 Materials and methods

### 2.1 Taxon sampling and data collection

A total of 49 species representing nine tribes in three subfamilies of Rosaceae was sampled (**Table 1**), including one genus in Dryadoideae, two genera in Rosoideae, and 46 genera in Amygdaloideae. The tribes in Amygdaloideae were well represented with three genera in Exochordeae, two genera in Kerrieae, two genera in Neillieae, one genus in Sorbarieae, three genera in Spiraeeae, and 35 genera in Maleae. DNA was deposited in the Plant DNA Bank of China and associated specimens in the PE National Specimen Resources Bank.

**Table 1:** Taxa in Rosaceae that sampled for chloroplast genome determinations with voucher information.

	Subfamily	Tribe	Taxon	Locality	Vocher (PE)
1	Amygdaloideae	Exochordeae	*Exochorda racemosa* (Lindl.) Rehder	Beijing Botanical Garden, CAS	BOP010047
2	Amygdaloideae	Exochordeae	*Oemleria cerasiformis* (Torr. & A. Gray) J. W. Landon	Royal Botanic Gardens, Kew	BOP040569
3	Amygdaloideae	Exochordeae	*Prinsepia sinensis* (Oliv.) Oliv. ex Bean	Beijing Botanical Garden, CAS	PGP00060
4	Amygdaloideae	Kerrieae	*Kerria japonica* (L.) DC.	Beijing Botanical Garden, CAS	PGP00063
5	Amygdaloideae	Kerrieae	*Rhodotypos scandens* (Thunb.) Makino	Beijing Botanical Garden, CAS	PGP00064
6	Amygdaloideae	Maleae	*Gillenia trifoliata* (L.) Moench	Royal Botanic Gardens, Kew	BOP022159
7	Amygdaloideae	Maleae	*Lindleya mespiloides* Schltdl.	PE (1683749)	BOP022161
8	Amygdaloideae	Maleae	*Vauquelinia corymbosa* Bonpl.	PE (1683677)	BOP022177
9	Amygdaloideae	Maleae	*Amelanchier sinica* (C. K. Schneid.) Chun	Beijing Botanical Garden, CAS	BOP027863
10	Amygdaloideae	Maleae	*Aria nivea* Host	PE (1498816)	BOP022174
11	Amygdaloideae	Maleae	*Aronia arbutifolia* (L.) Pers.	Shanghai Chenshan Botanical Garden,CAS	PGP00055
12	Amygdaloideae	Maleae	*Chaenomeles speciosa* (Sweet) Nakai	Beijing Botanical Garden, CAS	BOP010027
13	Amygdaloideae	Maleae	*Chamaemespilus humilis* (Lam.) M. Roem.	PE (1683185)	BOP022175
14	Amygdaloideae	Maleae	*Cotoneaster multiflorus* Bunge	Beijing Botanical Garden, CAS	BOP010016
15	Amygdaloideae	Maleae	*Crataegus kansuensis* E. H. Wilson	Beijing Botanical Garden, CAS	BOP010010
16	Amygdaloideae	Maleae	*Cydonia oblonga* Mill.	Beijing Botanical Garden, CAS	BOP010020
17	Amygdaloideae	Maleae	*Dichotomanthes tristaniicarpa* Kurz	Kunming Institute of Botany, CAS	BOP027700
18	Amygdaloideae	Maleae	*Docynia delavayi* (Franch.) C. K. Schneid.	Kunming Institute of Botany, CAS	BOP027851
19	Amygdaloideae	Maleae	*Eriolobus doumeri* (Bois) C. K. Schneid. [*Docyniadoumeri* (Bois) C. K. Schneid. ]	Hangzhou Botanical Garden, Zhejiang	BOP214859
20	Amygdaloideae	Maleae	*Docyniopsis tschonoskii* (Maxim.) Koidz.	PE (1702134)	BOP022164
21	Amygdaloideae	Maleae	*Eriobotrya japonica* (Thunb.) Lindl.	Beijing Botanical Garden, CAS	BOP003404
22	Amygdaloideae	Maleae	*Hesperomeles sp.*	South America	PGP00121
23	Amygdaloideae	Maleae	*Heteromeles arbutifolia* Greene	PE (1656086)	BOP022160
24	Amygdaloideae	Maleae	*Malus × spectabilis* (Sol.) Borkh.	Beijing Botanical Garden, CAS	BOP010021
25	Amygdaloideae	Maleae	*Eriolobus kansuensis* (Batalin) C. K. Schneid. [*Maluskansuensis* (Batalin) C. K. Schneid.]	Huludao, Liaoning	BOP011038
26	Amygdaloideae	Maleae	*Malus sieversii* (Ledeb.) M. Roem.	Beijing Botanical Garden, CAS	PGP00069
27	Amygdaloideae	Maleae	*Mespilus germanica* L.	PE (1765749)	BOP022165
28	Amygdaloideae	Maleae	*Micromeles alnifolia* (Siebold & Zucc.) Koehne	Beijing Botanical Garden, CAS	PGP00056
29	Amygdaloideae	Maleae	*Osteomeles schwerinae* C. K. Schneid.	Kunming Institute of Botany, CAS	BOP027697
30	Amygdaloideae	Maleae	*Peraphyllum ramosissimum* Nutt. ex Torr. & A.Gray	PE (1656430)	BOP022168
31	Amygdaloideae	Maleae	*Photinia integrifolia* Lindl.	Kunming Institute of Botany, CAS	BOP027699
32	Amygdaloideae	Maleae	*Photinia serratifolia* (Desf.) Kalkman	Beijing Botanical Garden, CAS	BOP027633
33	Amygdaloideae	Maleae	*Pourthiaea benthamiana* (Hance) Nakai	Bawangling, Hainan	BOP216344
34	Amygdaloideae	Maleae	*Pourthiaea hirsuta* (Hand.-Mazz.) Iketani & H. Ohashi	Wuyanling, Zhejiang	PGP00057
35	Amygdaloideae	Maleae	*Pseudocydonia sinensis* (Thouin) C. K. Schneid.	Beijing Botanical Garden, CAS	BOP010349
36	Amygdaloideae	Maleae	*Pyracantha fortuneana* (Maxim.) H. L. Li	Beijing Botanical Garden, CAS	BOP003403
37	Amygdaloideae	Maleae	*Pyrus bretschneideri* Rehder	Beijing Botanical Garden, CAS	BOP010065
38	Amygdaloideae	Maleae	*Rhaphiolepis indica* (L.) Lindl.	Kunming Institute of Botany, CAS	BOP016354
39	Amygdaloideae	Maleae	*Sorbus tianschanica* Rupr.	Kangfu, Xinjiang	BOP016989
40	Amygdaloideae	Maleae	*Stranvaesia davidiana* Decne. [*Photinia davidiana*(Decne.) Cardot]	Kunming Institute of Botany, CAS	BOP027698
41	Amygdaloideae	Neillieae	*Neillia thyrsiflora* D. Don	Xichou County, Yunnan	PGP00075
42	Amygdaloideae	Neillieae	*Physocarpus amurensis* (Maxim.) Maxim.	Beijing Botanical Garden, CAS	PGP00079
43	Amygdaloideae	Sorbarieae	*Sorbaria sorbifolia* (L.) A. Braun	Haerbin, Heilongjiang	BOP016568
44	Amygdaloideae	Spiraeeae	*Aruncus sylvestris* Kostel.	Qixiashan Botanical Garden,Heilongjiang	BOP016706
45	Amygdaloideae	Spiraeeae	*Sibiraea angustata* (Rehder) Hand.-Mazz.	Zuogong, Tibet	BOP017921
46	Amygdaloideae	Spiraeeae	*Spiraea pubescens* Turcz.	Beijing Botanical Garden, CAS	BOP010042
47	Dryadoideae	Dryadeae	*Dryas octopetala* L.	Ny-Ålesund, Svalbard, Arctic	BOP017859
48	Rosoideae	Roseae	*Rosa rugosa* Thunb.	Beijing Botanical Garden, CAS	BOP010536
49	Rosoideae	Rubeae	*Rubus palmatus* Thunb.	Beijing Botanical Garden, CAS	BOP010130

A total of 81 chloroplast genomes from an equal number of species, representing 69 genera in all three subfamilies (three genera of Dryadeae in Dryadoideae; 12 genera of Agrimonieae, two genera of Colurieae, and 10 genera of Potentilleae in Rosoideae; and one genus of Amygdaleae, two genera of Kerrieae, one genus of Lyonothamneae, 30 genera of Maleae, two genera of Sorbarieae, and five genera of Spiraeeae in Amygdaloideae), was downloaded from the GenBank (**Supplementary Table S2**).

In total, the newly determined chloroplast genomes (**Table 1**) along with those available in the GenBank (**Supplementary Table S2**) represented most tribes in Rosaceae, all 8 tribes in Amygdaloideae, and 35 out of 36 genera in Maleae were included in this study. However, we were unable to obtain material from *Chamaemeles*, a monotypic genus of South America, as only three chloroplast fragments are deposited in GenBank, which are insufficient for identifying its taxonomic position.

### 2.2 DNA extraction and chloroplast genome sequencing

Total genomic DNA was extracted from silica-gel dried leaf materials using the mCTAB method (Li et al., 2013) and purified using the Wizard DNA Clean-Up System (Promega, Madison, WI, USA). Chloroplast genomes were amplified using the primers listed in **Supplementary Table S3**. PCR amplification was performed in a final volume of 20 μl, containing 1× Taq buffer (1 mol L^-1^ KCl; 20 mmol Tris-HCl, pH 9.0; and 1% Triton X-100), 2.0 μl dNTPs (2 mmol L^-1^), 1.0 μl of each primer (5 μmol L^-1^), 20 ng of genomic DNA, and 1 unit of *Taq* polymerase. PCR was carried out in a C1000 Thermal Cycler (Bio-Rad Laboratories, Hercules, CA, USA) as follows: initial denaturation at 94°C for 3 min, followed by 35 cycles denaturation at 94°C for 30 s, annealing at 50°C for 30 s, and elongation at 72°C for 10 min, and a final elongation step at 72°C for 5 min. The PCR products were purified with a 1:1 mixture of 40% PEG 8000 and 5 mol L^-1^ NaCl, followed by a washing step with 80% ethanol. The library construction and sequencing with the pair-end Hiseq PE 150 on the Illumina Xten platform were performed by Novogene (Chaoyang, Beijing).

### 2.3 Data preparation

Reads were cleaned by removing all low-quality paired-end reads. Clean reads were *de novo* assembled using SPAdes 3.9 (Bankevich et al., 2012), and the generated contigs were mapped to the closest references using BLASTn 2.8.10 (Altschul et al., 1990). The mapped contigs were assembled using Sequencher 5.4 (Gene Codes, Ann Arbor, MI, USA), and gaps were filled by Sanger sequencing in Sangon Biotech.

### 2.4 Genome splitting and homologous fragment alignment

The newly determined chloroplast genomes were annotated by Geseq (Tillich et al., 2017). The option “annotate plastid IR” was used to determine the boundary of invert-repeat regions. After a manual check, the annotated genomes along with those retrieved from the GenBank were split into fragments (coding vs. non-coding regions) using BarcodeFinder (https://github.com/wpwupingwp/BarcodeFinder). Homologous regions were grouped, and then, each one was aligned using MAFFT 7.408 (Katoh and Standley, 2013) and adjusted manually with Se-Al 2.0 (Rambaut, 1996).

### 2.5 Dataset preparation

The datasets were separated into three taxonomic categories to increase accuracy. At the family level, the dataset contained 88 genomes (all the representative members of tribes and one genome from each genus in Maleae); at the Maleae level, the dataset contained 65 genomes (one or two representative members from all genera); and at the core Maleae level, the dataset contained 59 genomes (one or two representative members from all genera except *Gillenia*, *Kageneckia*, *Lindleya*, and *Vauquelinia*). Genome data were concatenated according to coding and non-coding regions using SequenceMatrix (Vaidya et al., 2011). Sequences in the two inverted regions were only used once. The nucleotide substitution models for the coding and non-coding datasets were selected by MrModelTest (Nylander, 2004) using the Bayesian information criteria (BIC).

### 2.6 Phylogenetic analyses

All datasets were analyzed with PAUP 4.0b10 (Swofford, 2003) for maximum parsimony (MP), with RaxML-HPC2 on XSEDE 8.2.12 (Stamatakis, 2014). for maximum likelihood (ML), and with MrBayes on XSEDE 3.2.6 (Ronquist and Huelsenbeck, 2003) for Bayesian inference (BI). ML and BI analyses were performed on CIPRES (Miller et al., 2010).

MP analyses employed a heuristic search strategy of 10,000 replicates that treated all characters as equally weighted and unordered, obtaining the starting trees with stepwise addition, random stepwise addition of 100 replicates, tree-bisection-reconnection (TBR), and MulTrees enabled. Branch support for MP trees was assessed with 1,000 bootstrap replicates, and all trees were saved at each replicate.

The nucleotide substitution models were selected by ModelFinder using BIC. Candidate models were restricted to RaxML supported by the “-mset raxml” option for ML analysis or by the “-mset mrbayes” option for BI analysis. ML analysis with 1,000 nonparametric bootstrap (BP) replicates was performed using the best-fit model.

Default settings were used for MrBayes; 2× four chains were run for 100,000,000 generations and sampled every 1,000 generations. Posterior probabilities (PP) were calculated from almost all the sampled trees when the standard deviation of the split frequencies permanently fell below 0.01. The trees sampled during the burn-in phase were discarded.

## 3 Results

### 3.1 Chloroplast genome features

The dataset used in this study contained 49 newly determined chloroplast genomes (**Table 1**) as well as 81 chloroplast genomes downloaded from GenBank (**Supplementary Table S2**), representing 117 species from 88 genera in Rosaceae. The general genome features of the 49 newly determined chloroplast genomes were presented in **Table 2**.

**Table 2:** Features of 49 chloroplast genomes of Rosaceous species.

Item	Feature								
	Dryadoieae	Rosoideae	Amygdaloideae					
			Maleae	Exochordeae	Kerrieae	Neillieae	Sorbarieae	Spiraeeae
Genome size (kbp)	158,310	155,905~156,567	152,226~161,738	159,043~160,568	158,334~159,695	158,907~159,212	159,222	155,821~160,124
Length of LSC (kbp)	86,962	85,341~85,880	80,351~89,930	87,293~87,316	86,294~87,481	87,324~87,607	88,943	84,293~86,060
Length of SSC (kbp)	18,478	18,720~19,033	18,878~19,466	19,182~19,476	19,116~19,457	18,817~18,835	17,433	18,804~21,378
Length of IR (kbp)	26,435	25,922~25,935	26,241~26,460	26,250~26,395	26,383~26,462	26,384~26,385	26,423	26,256~26,362
CG content (%)	36.5	37.1~37.3	36.4~36.9	36.5~36.7	36.3~36.9	36.3~36.5	36.1	36.4~36.8
Total number of genes	112	112	112	112	112	112	112	112
Protein-coding genes	78	78	78	78	78	78	78	78
tRNA genes	30	30	30	30	30	30	30	30
rRNA genes	4	4	4	4	4	4	4	4
Genes with introns	17	17	17	17	17	17	17	17

The chloroplast genome of Maleae showed a typical structure (**Supplementary Figure S1**): a circular double-stranded structure with two short inverted repeat (IRa and IRb) regions separated by a large single copy (LSC) region and a small single-copy (SSC) region. The genome size ranged from 155,367 bp to 159,695 bp, whereas the overall GC content was 36.35–37.23%. No significant differences were found in the chloroplast genome size, the overall GC content, or the size of each region within the Maleae but only among the subfamilies in Rosaceae.

A total of 113 coding genes was found in the chloroplast genome of Maleae, of which 79 were protein-coding genes, 30 were distinct tRNA genes, and four were rRNA genes (16S, 23S, 5S, and 4.5S). Based on their functions, the genes were divided into three categories: I) 60 genes related to transcription and translation, including subunits of RNA polymerase, rRNA, and ribosomal proteins (most of which were tRNA genes); II) 47 genes related to photosynthesis, including Rubisco large subunit genes, genes of various components in the photosynthetic electron transport chain, and genes presumed to be NAD(P)H dehydrogenase subunits; and III) six genes related to the biosynthesis of amino acids, fatty acids, and other substances, as well as some genes with unknown functions. Among the 113 genes, 19 genes contained introns: 17 contained one intron, whereas *ycf*3 and *clp*P contained two introns. The gene *rps*12 was a special trans-splicing gene with the 5’-terminal exon in LSC and the 3’-terminal exon in the IR region.

### 3.2 Variability of chloroplast genomes

A total of 83 coding regions and 267 noncoding regions (introns and intergenic spacers) were identified. Variability in 162 regions (>100 bp) of the 65 core Maleae chloroplast genomes was parameterized in percentages of variable sites [*p*, ratio of number of variable sites (S) to net length (nL); *p* = S/nL] and nucleotide diversity (π) (**Supplementary Table S4**). The top 10 variable regions were *ndh*C-*trn*V^uac^, *ndh*F-*rpl*32, *ndh*G-*ndh*I, *psb*Z-*rps*14, *rpl*33-*rps*18, *trn*G^gcc^-*trn*R^ucu^, *trn*H^gug^-*psb*A, *trn*R^ucu^-*atp*A, *trn*T^ugu^-*trn*L^uaa^, and *trn*W^cca^-*trn*P^ugg^. No variable sites were observed in the regions of *rpl*23-*ycf*2, *trn*A^ugc^, *trn*N^guu^-*ycf*1, and *trn*V^gac^-*rrn*16.

### 3.3 Systematic position of Maleae in Rosaceae

To reduce the computational burden, only one representative genome from each genus was selected for reconstructing the phylogeny of Rosaceae. All genera in the family were well represented, and the branch length of clades was a good indicator of the tribe rank. The pome-bearing genera formed a highly supported (bs = 100) clade and along with *Gillenia*, *Kageneckia*, *Lindleya*, and *Vauquelinia* composed the tribe Maleae (**Supplementary Figure S2**). The branch length of the clade was much longer than those within the clade. Six additional monophyletic clades in the subfamily Amygdaloideae corresponded to Lyonothamneae, Neillieae, Exochordeae, Kerrieae, Amygdaleae, Sorbarieae, and Spiraeeae. The latter was considered the sister tribe of Maleae.

### 3.4 Phylogenetic relationships within the tribe Maleae

Phylogenetic relationships among the major lineages within the tribe Maleae were fully determined (**Supplementary Figures S3**, **S4**). Four highly supported major lineages with relatively long branches were identified: (1) *Gillenia*, (2) *Lindleya* & *Kageneckia*, (3) *Vauquelinia*, and (4) the core Maleae. Thus, the monophyly of the former four genera as shown in **Supplementary Figure S2** was polished, whereas that of the core Maleae was highly supported by *Pyracantha* M. Roem. at the base. However, the genera in the core Maleae were very closely related, and in some cases, the systematic relationships were sensitive to data alignment.

### 3.5 Phylogenetic relationships within the core Maleae

**Figure 1:**
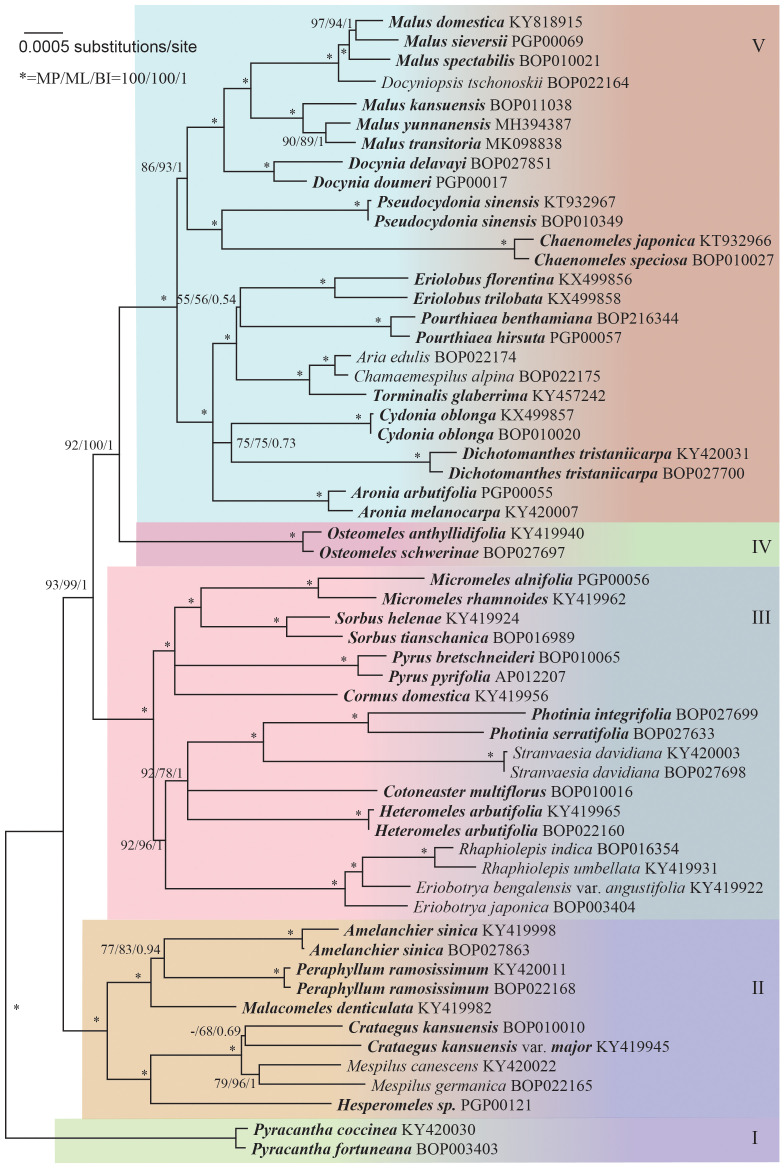
Maximum likelihood tree based on 59 complete chloroplast genomes, representing the phylogenetic relationships within the core Maleae. Supports to the branches are provided in the order of maximum parsimony bootstrap, maximum likelihood bootstrap, and Bayesian posterior probabilities. “-” indicates a branch collapse in the maximum parsimony and maximum likelihood trees. “*” indicates a branch with fully supports in MP/ML/BI. The tree was rooted using *Pyracantha* genomes as outgroups according to **Supplementary Figures S2**, **S3**.

The sequence data alignment of the core Maleae was carefully adjusted. The unreliable microsatellite regions were excluded from the analysis, resulting in a phylogenetic tree with well-supported clades (**Figure 1**). Five groups of lineages were indicated: Group I that consisted of *Pyracantha*; Group II that consisted of *Amelanchier*, *Crataegus*, *Hesperomeles* Lindl., *Malacomeles* (Decne.) Engl., *Mespilus*, and *Peraphyllum* Nutt.; Group III that consisted of *Cormus* Spach, *Cotoneaster* Medik., *Eriobotrya*, *Heteromeles* M.Roem., *Micromeles* Decne., *Rhaphiolepis*, *Photinia* Lindl., *Pyrus* L., *Sorbus* L., and *Stranvaesia* Lindl.; Group IV that consisted of *Osteomeles* Lindl.; and Group V that consisted of *Aria* (Pers.) Host, *Aronia* Medik., *Chaenomeles* Lindl., *Chamaemespilus* Medik., *Cydonia* Mill., *Dichotomanthes* Kurz, *Docynia* Decne., *Docyniopsis* (C. K. Schneid.) Koidz, *Eriolobus* (DC.) M. Roem., *Malus* Mill., *Pourthiaea* Decne., *Pseudocydonia* C. K. Schneid., and *Torminalis* Medik.

## 4 Discussion

### 4.1 Systematic position of *Gillenia*

After the merge of Maloideae with Amygdaloideae, it seemed reasonable to also merge the pome-bearing taxa with the non-pome-bearing taxa. The inclusion of *Kageneckia*, *Lindleya*, and *Vauquelinia* in the Maleae cannot be considered controversial as they are all tetraploids with the same basal chromosome number (x = 15 or 17), similarly as the core Maleae. The diploid *Gillenia* (x = 9) has been either placed in the distinct tribe Gillenieae (i.e., Angiosperm Phylogeny Website; https://www.mobot.org) or at an uncertain systematic position (i.e., National Center for Biotechnology Information; https://www.ncbi.nlm.nih.gov/Taxonomy). Although *Gillenia* diverged earlier than *Kageneckia*, *Lindleya*, *Vauquelinia*, and the core Maleae, the branch length of the clade was long enough to include all of them in the same tribe (Xiang et al., 2016, Figure 2; Zhang et al., 2017, Figure 1). It would be trivial to create a new tribe that included only two species from the same genus. In Amygdaloideae, some tribes and genera diverged in the Cretaceous but the divergence of *Gillenia* from the other members of Maleae probably occurred in the Eocene (Xiang et al., 2016, Figure 4). The inclusion of *Gillenia* in Maleae explained the origin of the tetraploid taxa from ancestral paleo-allotetraploid maternal parents.

### 4.2 Subdivision of Maleae

A hypothetical subdivision of Maleae would create four natural groups: Group A that would include *Gillenia*, a genus with the most basal position and ancestors of all other members; Group B that would include *Kageneckia* and *Lindleya*, two genera that form a well-supported clade and share some apomorphic morphological characters such as dry and dehiscent fruits; Group C that would include *Vauquelinia*, a genus of two North American species that differs from other members in the basal chromosome number (x = 15 instead of x = 9 or 17); and Group D that would include the pome-bearing genera or the core Maleae. Thus, the tribe could be subdivided into four subtribes: Gilleniinae (*Gillenia*), Lindleyinae (*Kageneckia* and *Lindleya*), Vauqueliniinae (*Vauquelinia*), and Malinae (the core Maleae).

### 4.3 Merging or splitting intractable genera in core Maleae

The phylogeny of the core Maleae remained unclear owing to the low resolution and incongruence of used molecular markers as well as to paralog problems (Campbell et al., 2007). Despite the controversies, especially at the generic level (Robertson et al., 1991), we supported the existence of five groups (**Figure 1**). Since there was only one genus each in Group I and IV, disputes were focused on Groups II, III and V.

In Group II, *Crataegus* and *Mespilus* were monophyletic but also extremely closely related. Besides, *Mespilus* included only two species, *M. canescens* (triploid) and *M. germanica* (diploid). Eugenia et al. (2007); Lo and Donoghue (2012) showed that *Mespilus* was nested within a clade that mostly consisted of *Crataegus* species and consequently, merged the two genera.

In Group III, *Rhaphiolepis* was nested within *Eriobotrya*, making the latter paraphyletic. *Eriobotrya* and *Rhaphiolepis* have a similar morphology, which indicates their close relationship (Robertson et al., 1991). Besides, *Rhaphiolepis* is not reproductively isolated from *Eriobotrya* species since hybrids have been reported between the two genera (Aldasoro et al., 2005; Li et al., 2016). Our result supported the merge of *Eriobotrya* with *Rhaphiolepis*, which was consistent with Liu et al. (2020).

Species in *Stranvaesia* are morphologically similar to *Photinia*. Besides, *S. davidiana* is considered a member of *Photinia* (Liu et al., 2019). However, the close relationship between *Stranvaesia* and *Cotoneaster* that previously suggested by Campbell et al. (2007) and Sun et al. (2018) was not confirmed in the present study.

The phylogenetic relationships among the members of *Sorbus s. l.* have been previously reviewed by Sennikov and Kurtto (2017). Here, the splitting of *Sorbus s. l.* was necessary because some members belonged to Group III while others to Group V. The pinnately compound-leaved *Cormus*, *Sorbus s. str.*, and the simple-leaved *Micromeles* remained in Group III.

In Group V, the close phylogenetic relationship among *Aria*, *Chamaemespilus*, and *Torminalis* that was previously suggested by Campbell et al. (2007) and Lo and Donoghue (2012) was also confirmed in the present study. In contrast to previous studies (Campbell et al., 2007; Potter et al., 2007), our clades were highly supported and had short branches, indicating low phylogenetic divergence. Only the stem branch was long, revealing a relatively long evolutionary history. It was reasonable to merge them into one genus.

The genus *Malosorbus* was first proposed by Browicz in 1970 to justify the hybrid origin of *Malosorbus florentina* (or *Malus florentina*), which was later defined as a true species that occurs in many European countries (Schneider, 1906; Huckins, 1972; Qian et al., 2008). In the present study, *Malosorbus florentina* and *Malus trilobata* (or correctly *Eriolobus trilobata*) formed a clade that had a close relationship with *Pourthiaea* but not with *Torminalis* or *Malus*. Considering the unique systematic positions of *Malosorbus florentina* and *Malus trilobata*, *Eriolobus* was adopted to host the two species; however, the former should be renamed to *Eriolobus florentina* (Zuccagni) Stapf.

Phipps et al. (1990) reported that the genus *Malus* consists of sect. *Malus*, sect. *Sorbomalus*, sect. *Chloromeles* (North American species), sect. *Eriolobus*, and sect. *Docyniopsis* (East Asian species). The present study supported the inclusion of *Docyniopsis* but not of *Eriolobus*. Originally, sect. *Eriolobus* included only *M. trilobata* (= *Eriolobus trilobata*) for the eastern Mediterranean; however, our data indicated that the North American species *Malus florentina* (= *Eriolobus florentina*) was closely related to *E. trilobatus*.

### 4.4 Systematic position of *Chamaemeles*

The systematic position of *Chamaemeles* remains uncertain owing to the lack of chloroplast genome data. The sequences of three available genes suggested that it might belong to Group III; however, its exact position remains to be confirmed.

### 4.5 Taxonomic implications

All genera presented in the current study are widely accepted, and the clades are well-supported, considering the complexity of their origins. Our objective was to provide additional data that would shed light on the taxonomy of Maleae and especially on the core Maleae. We suggested that the tribe could be subdivided into four subtribes with all pome-bearing species in Malinae (the core Maleae) and also that the five groups in the core Maleae could be given a subtribal rank. However, additional research is needed to confirm the systematic position of *Chamaemeles*.

Chloroplast genome sequences are very helpful to clarify the maternal origin of species and identify their systematic position. However, two-copy nuclear genes are necessary for revealing the bi-parental origin of Maleae species and confirm their taxonomy.

## Data availability statement

The datasets presented in this study can be found in online repositories. The names of the repository/repositories and accession number(s) can be found below: NCBI (https://www.ncbi.nlm.nih.gov) with accession numbers: OK375413 to OK375461.

## Author contributions

JS: Writing – original draft, Writing – review & editing. DZ: Writing – review & editing. PQ: Writing – review & editing. YW: Investigation, Methodology, Resources, Writing – review & editing. PW: Methodology, Software, Writing – review & editing. KW: Investigation, Resources, Writing – review & editing. LG: Project administration, Supervision, Visualization, Writing – review & editing. LH: Funding acquisition, Supervision, Visualization, Writing – review & editing. SZ: Conceptualization, Funding acquisition, Supervision, Writing – review & editing.

## Supplementary material

Supplementary Figure 1 Chloroplast genome map of *Crataegus kansuensis*. Genes inside and outside the circle are transcribed clockwise and counterclockwise, respectively. Genes of different functional groups are shown in different colors. Thick lines indicate the extent of inverted repeats (IRa and IRb) that separate the genomes into small single copy (SSC) and large single copy (LSC) regions.

Supplementary Figure 2 Maximum parsimonious tree of 88 chloroplast genomes, showing the phylogenetic relationships within the Rosaceae and the systematic position of Maleae. The tree was rooted using Dryadoideae as an outgroup. Bootstrap support is 100% unless otherwise indicated. “*” indicates the branch of core Maleae.

Supplementary Figure 3 Maximum likelihood tree of 88 chloroplast genomes, showing the phylogenetic relationships within the Rosaceae and the systematic position of Maleae. The tree was rooted using Dryadoideae as an outgroup. Bootstrap support value is 100% unless otherwise indicated, and the branch support value below 50 were collapsed. “*” indicates the branch of core Maleae.

Supplementary Figure 4 Maximum likelihood tree of 65 chloroplast genomes, showing the phylogenetic relationships within the Maleae. The tree was rooted using *Gillenia* genome as an outgroup.

Supplementary Figure 5 Maximum parsimonious tree of 65 chloroplast genomes, showing the phylogenetic relationships within the Maleae. The tree was rooted using *Gillenia* genome as an outgroup.

Supplementary Table 1 Thirty-six generally accepted genera in Maleae.

Supplementary Table 2 Taxa of chloroplast genomes downloaded from the GenBank and used in phylogenetic analysis.

Supplementary Table 3 Primers used to amplify the chloroplast genomes of Rosaceae.

Supplementary Table 4 Variability of 162 regions of 59 chloroplast genomes of species in the core Maleae.
