# Supplementary figures and images for: Phylogeny of genera in Maleae (Rosaceae) based on chloroplast genome analysis
Source: Front Plant Sci. 2024 Mar 26;15:1367645. doi: 10.3389/fpls.2024.1367645 (PMC11002139; doi:10.3389/fpls.2024.1367645)

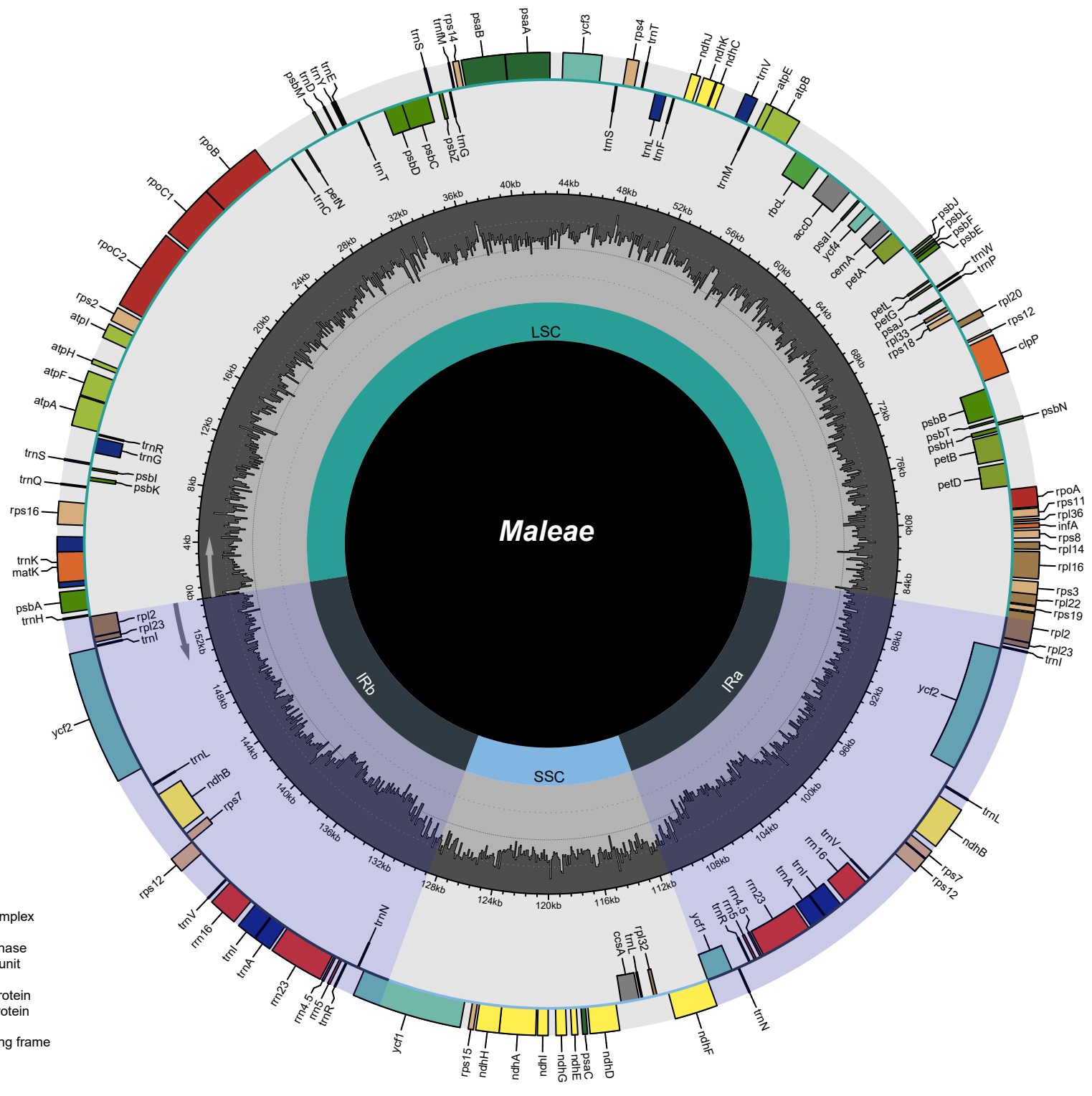

Supplement: Supplementary Figure 1 — Chloroplast genome map of Crataegus kansuensis. Genes inside and outside the circle are transcribed clockwise and counterclockwise, respectively. Genes of different functional groups are shown in different colors. Thick lines indicate the extent of inverted repeats (IRa and IRb) that separate the genomes into small single copy (SSC) and large single copy (LSC) regions. [file Image_1.pdf]

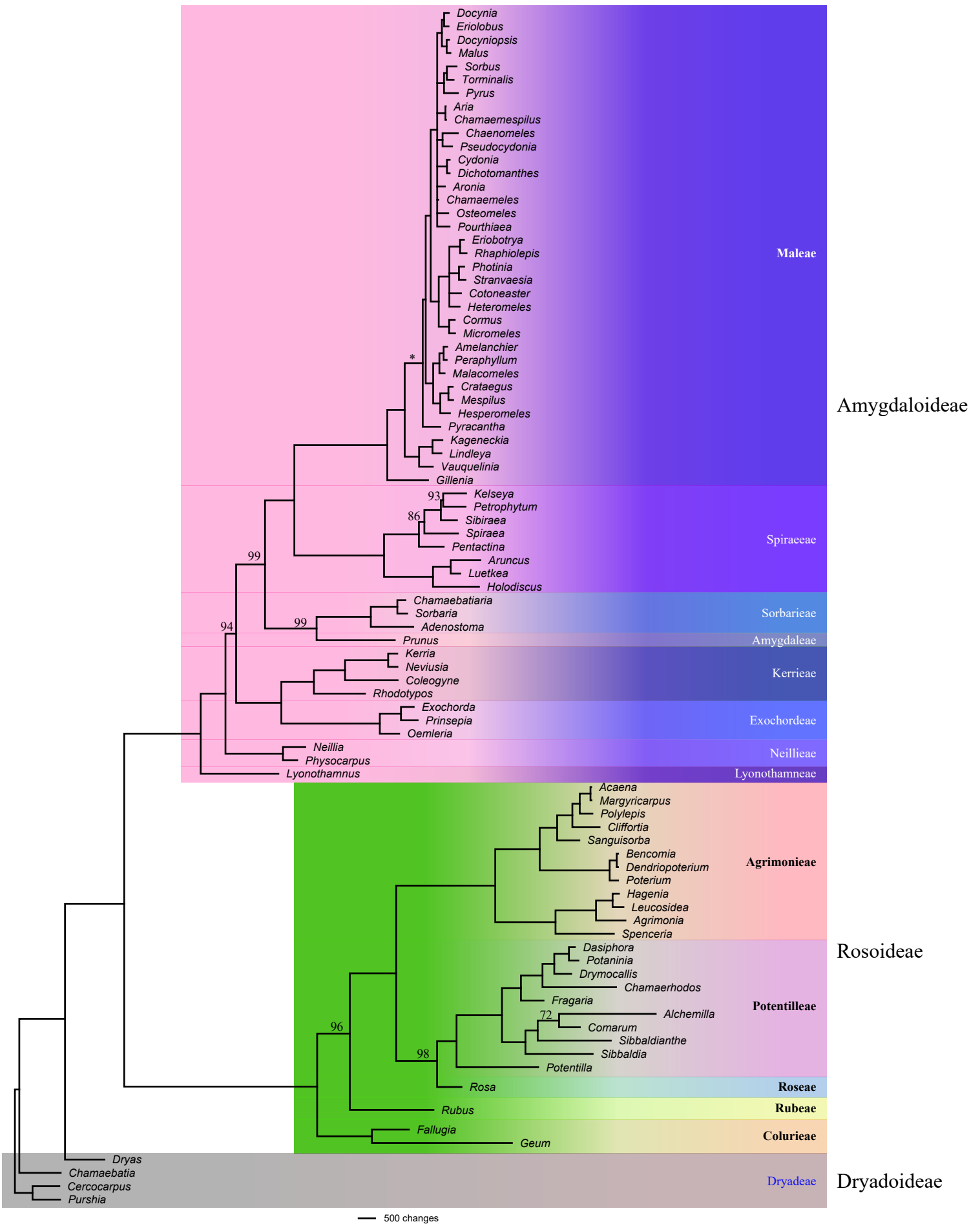

Supplement: Supplementary Figure 2 — Maximum parsimonious tree of 88 chloroplast genomes, showing the phylogenetic relationships within the Rosaceae and the systematic position of Maleae. The tree was rooted using Dryadoideae as an outgroup. Bootstrap support is 100% unless otherwise indicated. “*” indicates the branch of core Maleae. [file Image_2.pdf]

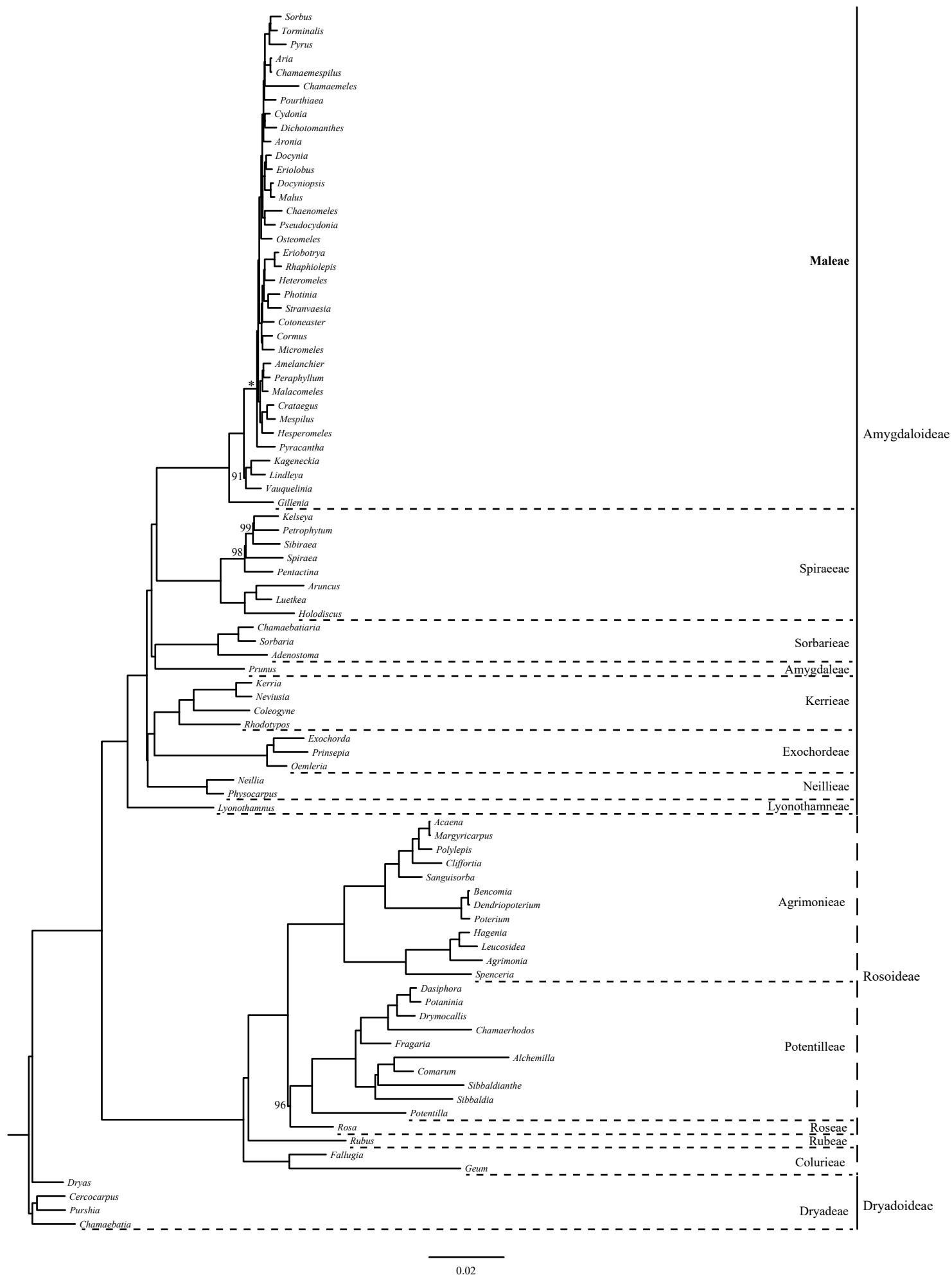

Supplement: Supplementary Figure 3 — Maximum likelihood tree of 88 chloroplast genomes, showing the phylogenetic relationships within the Rosaceae and the systematic position of Maleae. The tree was rooted using Dryadoideae as an outgroup. Bootstrap support value is 100% unless otherwise indicated, and the branch support value below 50 were collapsed. “*” indicates the branch of core Maleae. [file Image_3.pdf]

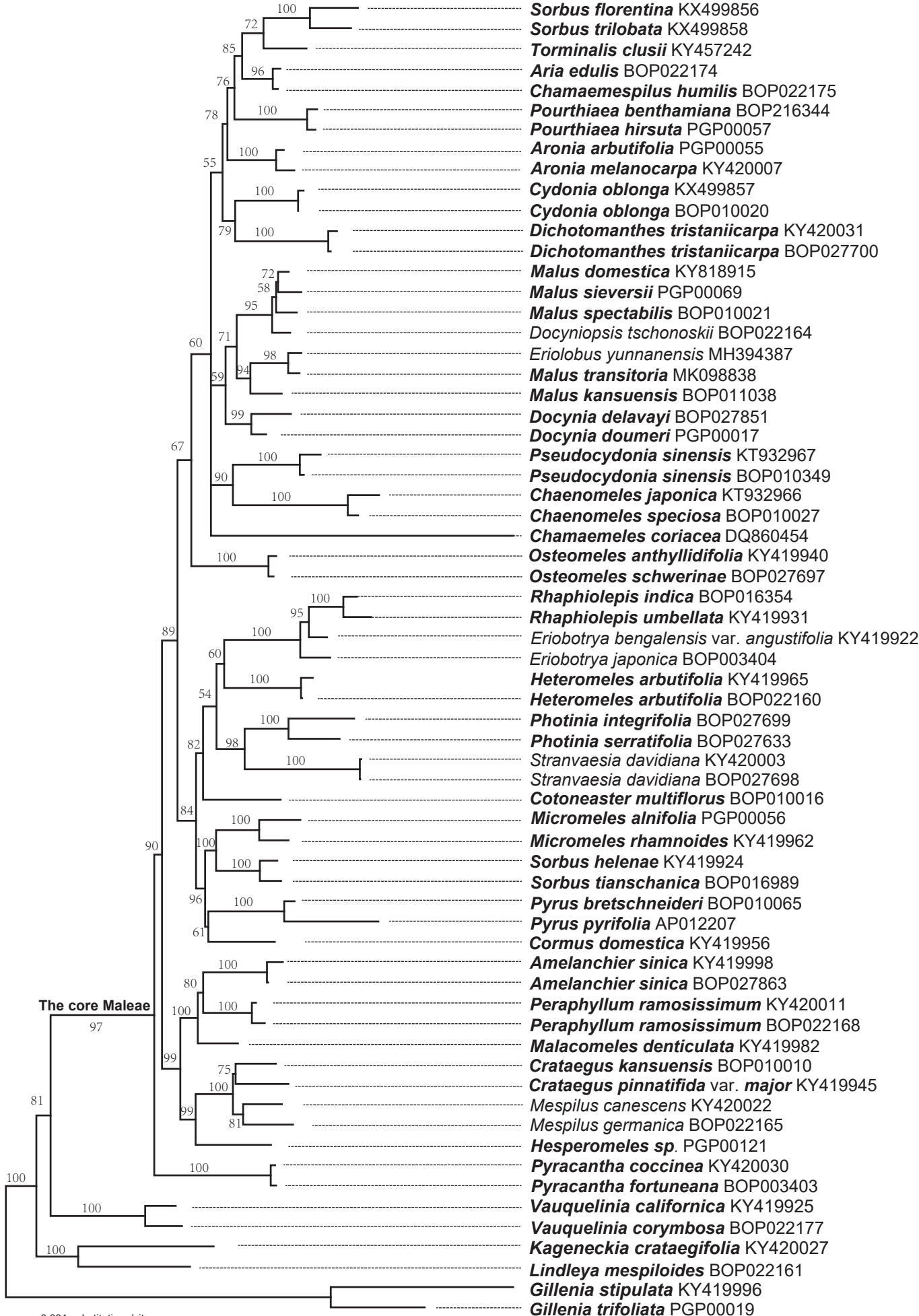

Supplement: Supplementary Figure 4 — Maximum likelihood tree of 65 chloroplast genomes, showing the phylogenetic relationships within the Maleae. The tree was rooted using Gillenia genome as an outgroup. [file Image_4.pdf]

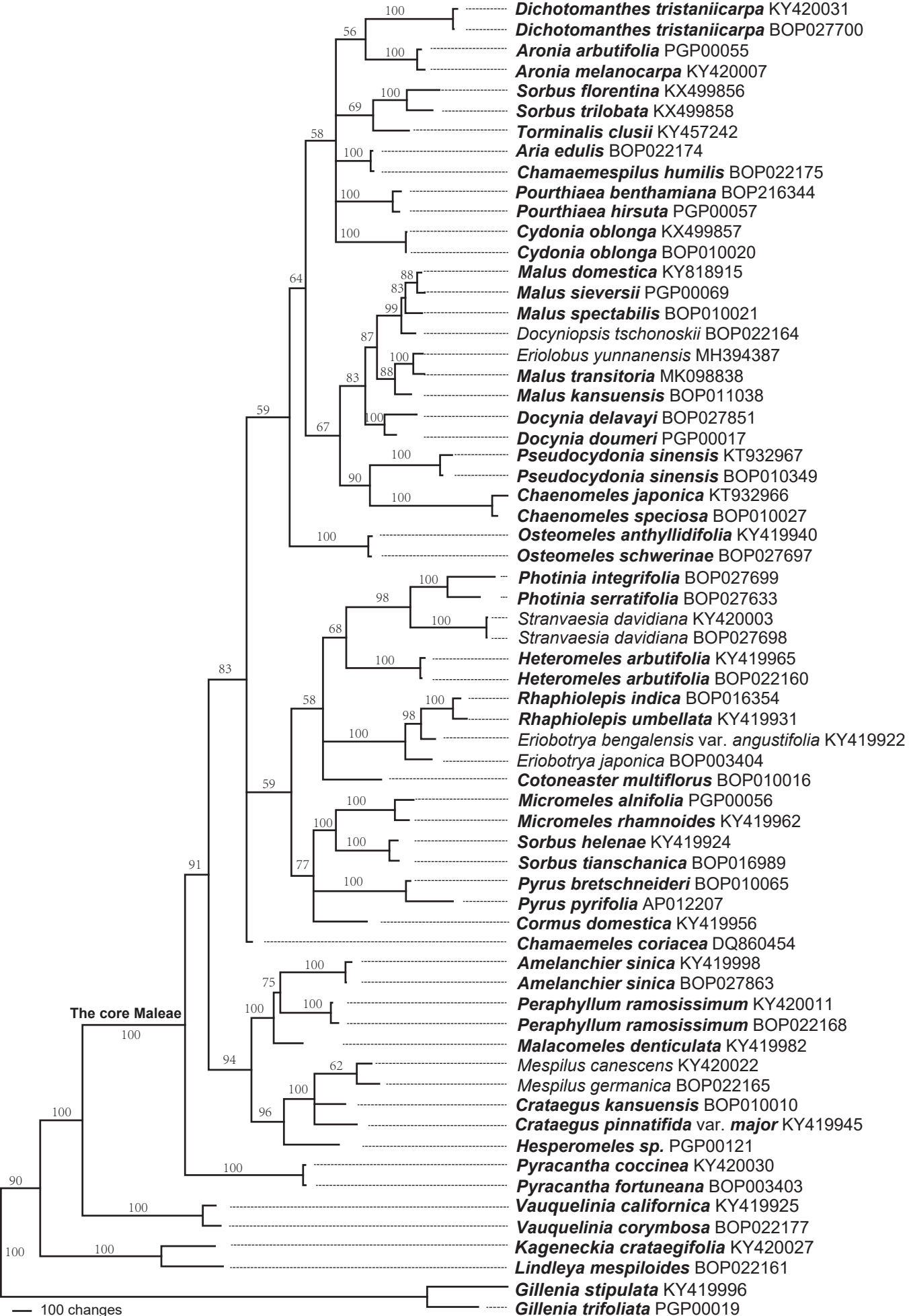

Supplement: Supplementary Figure 5 — Maximum parsimonious tree of 65 chloroplast genomes, showing the phylogenetic relationships within the Maleae. The tree was rooted using Gillenia genome as an outgroup. [file Image_5.pdf]
