# Supplementary material for: Phylogeny of genera in Maleae (Rosaceae) based on chloroplast genome analysis
Source: Front Plant Sci. 2024 Mar 26;15:1367645. doi: 10.3389/fpls.2024.1367645 (PMC11002139; doi:10.3389/fpls.2024.1367645)
Supplement: Supplementary Table 1 — Thirty-six generally accepted genera in Maleae. [file Table_1.pdf]

Table S1. Thirty-six generally accepted genera in Maleae

Maleae J. K. Small, Manual of the Southeaster Flora 632, 1933. Type: *Malus* Mill.

= Pyreae H. E. Baillon, Histoire des Plantes 1:442, 475, 1869.

= Gillenieae C. J. Maximowicz, Trudy Imperatorskago S.-Peterburgskago Botaničeskago Sata 6(1):164, 222, 1879.

Genus	Type	Group
Amelanchier Medikus in Philos. Bot. 1:135, 155, 1789.	*A. ovalis* Medic. = *Mespilus amelanchier* L.	II
Aria (Persoon) Host, Fl. Austriac. 2:7-8, 1831.	not designated	V
Aronia Medikus in Philos. Bot. 1: 140, 155, 1789.	*A. arbutifolia* = *Mespilus arbutifolia* L.	V
Chaenomeles Lindley in Trans. Linn. Soc. London 13(1):97, 1821, nom. cons.	*C. japonica* (Thunb.) Lindl. ex Spach. = *Pyrus japonica* Thunb.	V
Chamaemeles Lindley in Trans. Linn. Soc. London 13(1):96, 1821.	*C. coriacea* Lindley	V
Chamaemespilus Medikus in Philos. Bot. 1:138, 155, 1789.	*C. alpina* (Miller) Robertson and Phipps.	V
Cormus Spach, Hist. Nat. Veg. 2:96, 1834.	*C. domestica* Spach.	III
Cotoneaster Medikus in Philos. Bot. 1:154, 1789.	*C. integerrimus* Medic. = *Mespilus cotoneaster* L.	III
Crataegus L, Sp. Pl. 1:475, 1753.	*C. oxyacantha* L.	II
Cydonia Miller, Gard. Dict. Abr. (ed. 4) 1: Cydonia, 1754.	*C. oblonga* Miller =*Pyrus cydonia* L.	V

Dichotomanthes Kurz in J. Bot. 11(7):194-195, 1873.	*D. tristaniaecarpa* Kurz	V
Docynia Decaisne in Nouv. Ann. Mus. Hist. Nat. 10:125, 131, 1874.	*D. indica* (Wall.) Dcne. = *Pyrus indica* Wall.	V
Docyniopsis (C. K. Schneider) Koidzumi in Acta Phytotax. Geobot. 3:162, 1934.	not designated	V
Eriobotrya Lindley in Trans. Linn. Soc. London 13(1):96,102, 1821.	*E. japonica* (Thunb.) Lindl.	III
Eriolobus (A. P. de Candolle) M. J. Roemer, Fam. Nat. Syn. Monogr. 3:104, 216, 1847.	*E. trilobata* (Labill. Ex Poir.) M. J. Roemer.	V
Gillenia Moench, Suppl. Meth. 286, 1802.	*G. trifoliata* (L.) Moench	
Hesperomeles Lindley in Edwards's Bot. Reg. 23 ad t. 1956, 1837.	*H. cordata* (Lindl.) Lindl.	II
Heteromeles M. J. Roemer, Fam. Nat. Syn. Monogr. 3:100, 105, 1847, nom. cons.	*H. arbutifolia* M. Roemer.	III
Kageneckia Ruiz & Pav., Fl. Peruv. Prodr. 145, 1794.	*K. oblanga* Ruiz & Pav.	
Lindleya Kunth, Nov. Gen. Sp. (quarto ed.) 6: 239, 1824, nom. cons.	*L. mespiloides* Kunth	
Malacomeles (Decne.) Decne. in J. Gen. Hort. 23(7-9):156, 1880.	*M. denticulata* (Kunth) Decne.	II
Malus Miller, Gard. Dict. Abr. (ed. 4) 2: *Malus*, 1754.	*Malus sylvastris* Mill.	V
Mespilus L., Sp. Pl. 1:478, 1753.	*M. germanica* L.	II
Micromeles Decaisne in Nouv. Arch. Mus. Hist. Nat. 10: 125, 168, 1874.	not designated	III
Osteomele Lindley in Trans. Linn. Soc. London 13(1):96-99, 1821.	*O. anthyllidifolia* Lindl.	IV

Peraphyllum Nutt., Fl. N. Amer. 1(3):474, 1840.	*P. ramosissimum* Nuttall.	II
Photinia Lindley in Bot. Reg. 6: pl. 491, 1820, nom. cons.	*P. serrulata* Lindl.	III
Pourthiaea Decaisne in Nouv. Arch. Mus. Hist. Nat. 10: 125, 146, 1874.	not designated	V
Pseudocydonia C. C. in Repert. Spec. Nov. Regni Veg. 3(38/39):180, 1906.	*P. sinensis* (Thouin) C. K. Schneid.	V
Pyracantha M. J. Roemer, Fam. Nat. Syn. Monogr. 3:100, 104, 1847.	*P. coccinea* Roem. = *Mespilus pyracantha* L.	I
Pyrus L., Sp. Pl. 1:479, 1753.	*Pyrus communis* L.	III
Rhaphiolepis Lindley in Bot. Reg. 6:468, 1820, nom. cons.	*R. indica* (L.) Lindl. = *Crataegus indica* L.	III
Sorbus L., Sp. Pl. 1:477, 1753, nom. cons.	*S. aucuparia* L.	III
Stranvaesia Lindley, Edwards's Bot. Reg. 23: pl. 1956, 1837.	*S. glaucescens* Lindl. = *S. nussia* (Buch.-Ham.) Dcne.	III
Torminalis Medikus in Philos. Bot. 1:134, 155, 1789.	not designated	V
Vauquelinia Corrêa ex Bonpl., Pl. Aequinoct. 1(6): 140–142, pl. 40, 1807.	*V. corymbosa* Bonpl.	
