# Supplementary material for: Phylogeny of genera in Maleae (Rosaceae) based on chloroplast genome analysis
Source: Front Plant Sci. 2024 Mar 26;15:1367645. doi: 10.3389/fpls.2024.1367645 (PMC11002139; doi:10.3389/fpls.2024.1367645)
Supplement: Supplementary Table 2 — Taxa of chloroplast genomes downloaded from the GenBank and used in phylogenetic analysis. [file Table_2.pdf]

Table S2. Taxa of chloroplast genomes downloaded from the GenBank and used in phylogenetic analysis.

	Subfamily	Tribe	Taxon	Accession No.
1	Amygdaloideae	Amygdaleae	*Prunus salicina* Lindl.	KY420002.1
2	Amygdaloideae	Kerrieae	*Coleogyne ramosissima* Torr.	KY419967.1
3	Amygdaloideae	Kerrieae	*Neviusia cliftonii* Shevock, Ertter & D. W. Taylor	KY419980.1
4	Amygdaloideae	Lyonothamneae	*Lyonothamnus floribundus* A. Gray	KY420005.1
5	Amygdaloideae	Maleae	*Gillenia stipulata* (Muhl. ex Willd.) Nutt.	KY419996.1
6	Amygdaloideae	Maleae	*Kageneckia crataegifolia* Lindl.	KY420027.1
7	Amygdaloideae	Maleae	*Vauquelinia californica* (Torr.) Sarg.	KY419925.1
8	Amygdaloideae	Maleae	*Amelanchier sinica* (C. K. Schneid.) Chun	KY419998.1
9	Amygdaloideae	Maleae	*Aronia melanocarpa* (Michx.) Elliott	KY420007.1
10	Amygdaloideae	Maleae	*Chaenomeles japonica* (Thunb.) Lindl. ex Spach	KT932966.1
11	Amygdaloideae	Maleae	*Chamaemeles coriacea* Lindl.	DQ860454.1
12	Amygdaloideae	Maleae	*Cormus domestica* (L.) Spach	KY419956.1
13	Amygdaloideae	Maleae	*Crataegus pinnatifida* var. *major* N. E. Br.	KY419945.1
14	Amygdaloideae	Maleae	*Cydonia oblonga* Mill.	KX499857.1
15	Amygdaloideae	Maleae	*Dichotomanthes tristaniicarpa* Kurz	KY420031.1
16	Amygdaloideae	Maleae	*Docynia doumeri* (Bois) C. K. Schneid.	KX499861.1
17	Amygdaloideae	Maleae	*Docyniopsis tschonoskii* (Maxim.) Koidz.	KX499863.1
18	Amygdaloideae	Maleae	*Eriobotrya bengalensis* var. *angustifolia* Cardot	KY419922.1
19	Amygdaloideae	Maleae	*Eriobotrya japonica* (Thunb.) Lindl.	KT633951.1
20	Amygdaloideae	Maleae	*Eriolobus yunnanensis* (Franch.) C. K. Schneid.	MH394387.1
21	Amygdaloideae	Maleae	*Heteromeles arbutifolia* Greene	KY419965.1
22	Amygdaloideae	Maleae	*Malacomeles denticulata* (Kunth) G. N. Jones	KY419982.1
23	Amygdaloideae	Maleae	*Malus × prunifolia* (Willd.) Borkh.	KU851961.1
24	Amygdaloideae	Maleae	*Malus baccata* (L.) Borkh.	KX499859.1
25	Amygdaloideae	Maleae	*Malus domestica* Borkh.	KY818915.1
26	Amygdaloideae	Maleae	*Malus hupehensis* (Pamp.) Rehder	MK020147.1
27	Amygdaloideae	Maleae	*Malus micromalus* Makino	MF062434.1
28	Amygdaloideae	Maleae	*Malus transitoria* (Batalin) C. K. Schneid.	MK098838.1
29	Amygdaloideae	Maleae	*Mespilus canescens* J.B.Phipps	KY420022.1
30	Amygdaloideae	Maleae	*Micromeles alnifolia* (Siebold & Zucc.) Koehne	KY420010.1
31	Amygdaloideae	Maleae	*Micromeles rhamnoides* Decne.	KY419962.1
32	Amygdaloideae	Maleae	*Osteomeles anthyllidifolia* (Sm.) Lindl.	KY419940.1
33	Amygdaloideae	Maleae	*Peraphyllum ramosissimum* Nutt. ex Torr. & A.Gray	KY420011.1
34	Amygdaloideae	Maleae	*Photinia integrifolia* Lindl.	KY419933.1
35	Amygdaloideae	Maleae	*Photinia prionophylla* (Franch.) C. K. Schneid.	KY419946.1
36	Amygdaloideae	Maleae	*Pourthiaea arguta* var. *salicifolia* (Decne.) Hook. f.	KY419919.1
37	Amygdaloideae	Maleae	*Pseudocydonia sinensis* (Thouin) C. K. Schneid.	KT932967.1
38	Amygdaloideae	Maleae	*Pyracantha coccinea* M. Roem.	KY420030.1
39	Amygdaloideae	Maleae	*Pyrus pyrifolia* (Burm.f.) Nakai	AP012207.1
40	Amygdaloideae	Maleae	*Rhaphiolepis umbellata* (Thunb.) Makino	KY419931.1
41	Amygdaloideae	Maleae	*Eriolobus florentina* (Zuccagni) Stapf	KX499856.1
42	Amygdaloideae	Maleae	*Sorbus helenae* Koehne	KY419924.1
43	Amygdaloideae	Maleae	*Sorbus rufopilosa* C.K.Schneid.	KY419990.1
44	Amygdaloideae	Maleae	*Sorbus trilobata* (Labill. ex Poir.) Heynh.	KX499858.1
45	Amygdaloideae	Maleae	*Sorbus ulleungensis* Chin S. Chang	MG011706.1
46	Amygdaloideae	Maleae	*Stranvaesia davidiana* Decne.	KY420003.1
47	Amygdaloideae	Maleae	*Torminaria clusii* M. Roem.	KY457242.1
48	Amygdaloideae	Sorbarieae	*Adenostoma fasciculatum* Hook. & Arn.	KY387915.1
49	Amygdaloideae	Sorbarieae	*Chamaebatiaria millefolium* (Torr.) Maxim.	KY420017.1
50	Amygdaloideae	Spiraeeae	*Holodiscus discolor* (Pursh) Maxim.	KY420032.1
51	Amygdaloideae	Spiraeeae	*Kelseya uniflora* Rydb.	KY419988.1
52	Amygdaloideae	Spiraeeae	*Luetkea pectinata* (Pursh) Kuntze	KY419971.1
53	Amygdaloideae	Spiraeeae	*Pentactina rupicola* Nakai	JQ041763.1
54	Amygdaloideae	Spiraeeae	*Petrophytum caespitosum* (Nutt. ex Torr. & A. Gray) Rydb.	KY419970.1
55	Dryadoideae	Dryadeae	*Cercocarpus montanus* var. *minutiflorus* (Abrams) F. L. Martin	KY420024.1
56	Dryadoideae	Dryadeae	*Chamaebatia foliolosa* Benth.	KY419950.1
57	Dryadoideae	Dryadeae	*Purshia tridentata* (Pursh) DC.	KY420000.1
58	Rosoideae	Agrimonieae	*Acaena pinnatifida* Ruiz & Pav.	KY419984.1
59	Rosoideae	Agrimonieae	*Agrimonia pilosa* Ledeb.	KY419942.1
60	Rosoideae	Agrimonieae	*Bencomia exstipulata* Svent.	MG682353.1
61	Rosoideae	Agrimonieae	*Cliffortia repens* Schltr.	KY419983.1
62	Rosoideae	Agrimonieae	*Dendriopoterium menendezii* Svent.	KY419966.1
63	Rosoideae	Agrimonieae	*Hagenia abyssinica* (Bruce ex Steud.) J. F. Gmel.	KX008604.1
64	Rosoideae	Agrimonieae	*Leucosidea sericea* Eckl. & Zeyh.	KY419929.1
65	Rosoideae	Agrimonieae	*Margyricarpus pinnatus* (Lam.) Kuntze	KY419972.1
66	Rosoideae	Agrimonieae	*Polylepis reticulata* Hieron.	KY419921.1
67	Rosoideae	Agrimonieae	*Poterium spinosum* L.	KY419948.1
68	Rosoideae	Agrimonieae	*Sanguisorba officinalis* L.	KY419975.1
69	Rosoideae	Agrimonieae	*Spenceria ramalana* Trimen	KY419995.1
70	Rosoideae	Colurieae	*Fallugia paradoxa* (D. Don) Endl. ex Torr.	KY419999.1
71	Rosoideae	Colurieae	*Geum elatum* Wall. ex G. Don	KY419976.1
72	Rosoideae	Potentilleae	*Alchemilla pectinata* Kunth	KY419937.1
73	Rosoideae	Potentilleae	*Chamaerhodos erecta* (L.) Bunge	KY420001.1
74	Rosoideae	Potentilleae	*Comarum salesovianum* (Stephan) Asch. & Graebn.	KY420034.1
75	Rosoideae	Potentilleae	*Dasiphora fruticosa* (L.) Rydb.	MF683841.1
76	Rosoideae	Potentilleae	*Drymocallis glandulosa* (Lindl.) Rydb.	KY420015.1
77	Rosoideae	Potentilleae	*Fragaria orientalis* Losinsk.	KY769126.1
78	Rosoideae	Potentilleae	*Potaninia mongolica* Maxim.	KY419959.1
79	Rosoideae	Potentilleae	*Potentilla indica* (Jacks.) Th. Wolf	KY420014.1
80	Rosoideae	Potentilleae	*Sibbaldia procumbens* L.	KY419935.1
81	Rosoideae	Potentilleae	*Sibbaldianthe sericea* Grubov	KY419993.1
