# Supplementary material for: Phylogeny of genera in Maleae (Rosaceae) based on chloroplast genome analysis
Source: Front Plant Sci. 2024 Mar 26;15:1367645. doi: 10.3389/fpls.2024.1367645 (PMC11002139; doi:10.3389/fpls.2024.1367645)
Supplement: Supplementary Table 3 — Primers used to amplify the chloroplast genomes of Rosaceae. [file Table_3.pdf]

Table S3. Primers used to amplify the chloroplast genomes of Rosaceae.

Primer name	Forward (5'-3')	Reverse (5'-3')	Expected length (kbp)	Tm
ROS-CPG-1	GGGCGAACGACGGGAATTGAAC	ACGTCCAGGATTACGTCCTGGATCA	9	59/59
ROS-CPG-2	GCTGCTGTAAGTTTTCGATGATAT	TTTCTTTCAGAGGGTAATATGAATG	9	52/51
ROS-CPG-3	CGAATGAATTCAAGGACATATTC	GAACAACCTGTTATAATAGGAAAGC	9	50/53
ROS-CPG-4	ATTGTAGTACCAAGTACTTCTTG	GACATCTCTCTTTCAAGGAGGCAGC	9	50/59
ROS-CPG-5	TTGGGCCGAGCTGGATTTGAACC	TGGAATAATTTCTTAGATGTATTGC	9	59/49
ROS-CPG-6	GCAATAGCTAAATGATGATG	AGTCCGTAGCGTCTACCAATTTCGC	10	46/59
ROS-CPG-7	GATGACCATCGCATTACAAATGC	GGAAGGTATTGTCTATAATGATAGG	8	53/53
ROS-CPG-8	GTATCTACAGGACCTAAATTATC	TTACTCGTTAATGGTTGATCAAGTT	9	50/51
ROS-CPG-9	CTGGTCAGAAATATAGTGAAATC	TGATACTATGCAATTTGTGCGACC	9	50/54
ROS-CPG-10	AAATTCTCCCGTCTGTGCCTC	GTTATTCATGTTCAAGCAAGTTTC	8	54/51
ROS-CPG-11	GACGCTTACTGTCTGCTCTTGATTC	GCTTCTCACGTTCCGTGAATAGCCG	9	58/61
ROS-CPG-12	TCGTCAAATATTCAATATGATTCC	AGTTACTAATTCATGATCTGGCATG	9	49/53
ROS-CPG-13	CCATTCACTATTTCTTGAAGCTCG	GAGGTCATATCTAGTATTCAGAGTT	9	54/53
ROS-CPG-14	AAAGCGAGTCTTCATAGGGCAATTG	TTCACCATAGCGGCTTATTCGAAAT	9	56/54
ROS-CPG-15	TTCGTAAAAATATTTGGAAAAGGAA	TATGTGTGTTATCAATATCTCTACG	9	48/51
ROS-CPG-16	ATACTCTGGATTGGAATCGTG	CCAAGTAGGATTCGAACCTACGACC	9	50/59
