# Supplementary material for: Phylogeny of genera in Maleae (Rosaceae) based on chloroplast genome analysis
Source: Front Plant Sci. 2024 Mar 26;15:1367645. doi: 10.3389/fpls.2024.1367645 (PMC11002139; doi:10.3389/fpls.2024.1367645)
Supplement: Supplementary Table 4 — Variability of 162 regions of 59 chloroplast genomes of species in the core Maleae. [file Table_4.pdf]

Table S4. Variability of 162 regions of 59 chloroplast genomes of species in the core Maleae.

Region	Sequences	Sites	NetSites	S	S/NetSites	Hap	Hd	VarHd	Pi
accD	57	1482	1467	46	0.0314	34	0.9818	0.0000	0.0030
accD-psaI	55	1244	356	26	0.0730	18	0.7010	0.0048	0.0053
atpA	58	1524	1520	35	0.0230	31	0.9552	0.0002	0.0024
atpB	58	1505	1479	35	0.0237	28	0.9504	0.0002	0.0020
atpB-rbcL	56	821	728	34	0.0467	32	0.9643	0.0002	0.0036
atpE	57	402	402	12	0.0299	13	0.7682	0.0016	0.0029
atpF	58	1355	1253	50	0.0399	37	0.9812	0.0001	0.0032
atpF-atpH	56	577	400	21	0.0525	20	0.8552	0.0016	0.0049
atpH	58	246	246	5	0.0203	6	0.3406	0.0062	0.0015
atpH-atpI	56	1325	1045	94	0.0900	46	0.9935	0.0000	0.0088
atpI	58	750	744	18	0.0242	18	0.7913	0.0029	0.0018
atpI-rps2	56	246	212	13	0.0613	12	0.5604	0.0063	0.0046
ccsA	57	972	954	65	0.0681	35	0.9812	0.0000	0.0052
ccsA-ndhD	52	355	219	21	0.0959	22	0.8929	0.0010	0.0110
cemA	57	690	690	18	0.0261	19	0.7845	0.0031	0.0018
cemA-petA	55	256	197	17	0.0863	14	0.6545	0.0052	0.0069
clpP	56	2274	1908	110	0.0577	43	0.9890	0.0000	0.0042
clpP-psbB	56	539	442	25	0.0566	20	0.8734	0.0012	0.0043
infA	32	238	223	20	0.0897	17	0.7863	0.0060	0.0074
matK	57	1516	1501	108	0.0720	44	0.9919	0.0000	0.0064
matK-rps16	54	2028	1537	134	0.0872	45	0.9937	0.0000	0.0084
ndhA	57	2429	2079	120	0.0577	45	0.9919	0.0000	0.0054

ndhB	57	2202	2202	12	0.0054	8	0.3972	0.0063	0.0003
ndhB-rps7	58	326	315	8	0.0254	9	0.5094	0.0057	0.0021
ndhC	57	363	363	10	0.0275	10	0.5909	0.0055	0.0022
ndhC-atpE	30	2321	1493	84	0.0563	29	0.9977	0.0001	0.0059
ndhC-trnV[uac]	25	901	350	38	0.1086	18	0.9433	0.0013	0.0126
ndhD	57	1502	1501	71	0.0473	47	0.9931	0.0000	0.0047
ndhE	58	325	284	8	0.0282	9	0.4241	0.0067	0.0017
ndhE-ndhG	52	309	215	18	0.0837	20	0.7285	0.0046	0.0058
ndhF	57	2286	2198	132	0.0601	46	0.9925	0.0000	0.0053
ndhF-rpl32	52	1517	805	108	0.1342	46	0.9955	0.0000	0.0142
ndhG	57	531	531	16	0.0301	16	0.6805	0.0048	0.0020
ndhG-ndhI	52	469	335	37	0.1104	28	0.9600	0.0002	0.0134
ndhH	58	1182	1182	30	0.0254	25	0.8760	0.0016	0.0018
ndhI	58	497	497	14	0.0282	14	0.7780	0.0020	0.0027
ndhJ	58	477	477	11	0.0231	11	0.6939	0.0038	0.0019
ndhK	57	671	671	19	0.0283	18	0.7857	0.0031	0.0019
petA	58	963	963	23	0.0239	18	0.7447	0.0038	0.0016
petA-psbJ	56	1200	896	79	0.0882	42	0.9883	0.0000	0.0081
petB-petD	31	976	892	31	0.0348	25	0.9806	0.0002	0.0036
petD	23	1207	1190	28	0.0235	20	0.9842	0.0004	0.0032
petG-psaJ	30	1032	759	62	0.0817	26	0.9885	0.0002	0.0094
petN-psbM	55	1551	459	42	0.0915	35	0.9764	0.0001	0.0087
psaA	58	2253	2253	60	0.0266	43	0.9825	0.0001	0.0025
psaA-psabF	32	1890	1890	48	0.0254	28	0.9899	0.0001	0.0031

psaA-ycf3	26	828	627	26	0.0415	17	0.8862	0.0035	0.0042
psaaF-psaA	30	787	787	13	0.0165	10	0.6782	0.0088	0.0016
psaB	58	2205	2205	36	0.0163	25	0.9401	0.0004	0.0014
psaB-psaaF	32	1284	1284	18	0.0140	16	0.8548	0.0033	0.0014
psabF-ycf3	30	993	851	33	0.0388	20	0.8943	0.0028	0.0035
psaC	54	246	246	3	0.0122	4	0.1768	0.0048	0.0007
psaC-ndhE	49	264	245	11	0.0449	12	0.4915	0.0079	0.0026
psaI-ycf4	56	442	360	35	0.0972	25	0.8545	0.0020	0.0072
psaJ-rpl33	55	564	375	33	0.0880	29	0.9300	0.0006	0.0062
psbA	57	1062	1062	22	0.0207	17	0.7343	0.0039	0.0015
psbA-matK	29	741	506	29	0.0573	24	0.9852	0.0002	0.0083
psbA-trnK^{uuu}	25	316	216	14	0.0648	16	0.9167	0.0020	0.0087
psbB	58	1527	1527	32	0.0210	26	0.8766	0.0016	0.0013
psbC	58	1093	1056	16	0.0152	16	0.7096	0.0043	0.0010
psbC-psbZ	30	774	620	44	0.0710	20	0.9471	0.0007	0.0083
psbC-trnS^{uga}	25	262	235	10	0.0426	10	0.7800	0.0042	0.0059
psbD	57	1369	1368	29	0.0212	30	0.9693	0.0001	0.0024
psbE	58	252	252	3	0.0119	4	0.1652	0.0042	0.0007
psbE-petL	55	1430	1137	82	0.0721	37	0.9805	0.0001	0.0059
psbH	58	225	225	6	0.0267	7	0.2868	0.0060	0.0016
psbH-petB	31	1015	818	48	0.0587	22	0.9204	0.0019	0.0055
psbI-atpA	31	3853	2010	163	0.0811	31	1.0000	0.0001	0.0092
psbJ-psbF	23	309	261	1	0.0038	2	0.0870	0.0061	0.0003
psbK-psbI	54	274	232	16	0.0690	15	0.6667	0.0053	0.0045

psbM-psbD	30	4858	3430	226	0.0659	30	1.0000	0.0001	0.0071
psbM-trnD^{guc}	25	1342	950	52	0.0547	24	0.9967	0.0002	0.0072
psbZ-rps14	30	1472	750	80	0.1067	25	0.9862	0.0002	0.0101
psbZ-trnG^{ucc}	19	812	355	31	0.0873	15	0.9415	0.0023	0.0112
rbcL	58	1428	1428	60	0.0420	43	0.9903	0.0000	0.0058
rbcL-accD	55	754	519	38	0.0732	30	0.9630	0.0002	0.0062
rpl14	57	369	369	11	0.0298	12	0.6510	0.0047	0.0024
rpl16	24	1477	927	51	0.0550	23	0.9964	0.0002	0.0089
rpl16-rps3	30	1267	1061	69	0.0650	29	0.9977	0.0001	0.0076
rpl2	58	1548	1497	11	0.0073	10	0.5203	0.0062	0.0007
rpl20	57	354	354	13	0.0367	12	0.7813	0.0013	0.0039
rpl20-rps12	53	822	742	34	0.0458	31	0.9659	0.0002	0.0041
rpl22	58	426	379	20	0.0528	20	0.7641	0.0036	0.0037
rpl23	58	282	282	7	0.0248	4	0.1942	0.0046	0.0012
rpl23-ycf2	33	407	327	0	0.0000	1	0.0000	0.0000	0.0000
rpl32-ccsA	29	2319	1320	130	0.0985	29	1.0000	0.0001	0.0110
rpl32-trnL^{uag}	23	1921	1230	98	0.0797	23	1.0000	0.0002	0.0102
rpl33	58	201	201	6	0.0299	8	0.3932	0.0063	0.0023
rpl33-rps18	56	410	178	22	0.1236	21	0.8877	0.0008	0.0126
rpl36-rps8	23	469	443	33	0.0745	22	0.9960	0.0002	0.0095
rpoA	56	1002	978	44	0.0450	31	0.9597	0.0003	0.0030
rpoB	57	3207	3207	81	0.0253	35	0.9812	0.0000	0.0021
rpoB-petN	30	2569	2013	139	0.0691	29	0.9977	0.0001	0.0082
rpoB-trnC^{gca}	24	1314	1058	63	0.0595	23	0.9964	0.0002	0.0071

rpoc1	58	2865	2711	87	0.0321	45	0.9921	0.0000	0.0027
rpoc2	57	4226	4111	139	0.0338	48	0.9944	0.0000	0.0028
rps11	57	417	417	9	0.0216	8	0.2920	0.0062	0.0010
rps12	25	922	913	4	0.0044	5	0.3000	0.0139	0.0004
rps12-rrn16	32	2061	1968	14	0.0071	11	0.5746	0.0110	0.0005
rps14	58	303	303	6	0.0198	7	0.2868	0.0060	0.0010
rps15	57	367	262	9	0.0344	10	0.3221	0.0066	0.0016
rps15-ycf1	38	513	398	39	0.0980	24	0.9275	0.0012	0.0087
rps16	57	1304	804	74	0.0920	39	0.9825	0.0001	0.0074
rps16-psbK	29	1739	1012	61	0.0603	28	0.9975	0.0001	0.0063
rps16-trnQ^{uug}	25	1064	546	33	0.0604	21	0.9867	0.0002	0.0078
rps18	58	303	303	4	0.0132	4	0.1954	0.0047	0.0009
rps18-rpl20	55	282	256	25	0.0977	23	0.8626	0.0019	0.0115
rps19	47	279	279	10	0.0358	11	0.5356	0.0077	0.0022
rps2	58	711	711	14	0.0197	14	0.6697	0.0048	0.0013
rps2-rpoc2	25	303	261	12	0.0460	11	0.8400	0.0034	0.0058
rps3	57	657	633	25	0.0395	20	0.8239	0.0022	0.0028
rps4	58	606	606	11	0.0182	11	0.4749	0.0066	0.0012
rps4-ndhJ	31	4226	3077	229	0.0744	31	1.0000	0.0001	0.0082
rps4-trnT^{ugu}	25	556	379	16	0.0422	17	0.9500	0.0009	0.0061
rps7	58	483	468	1	0.0021	2	0.0998	0.0027	0.0002
rps7-trnV^{gac}	23	2577	2536	18	0.0071	14	0.8814	0.0038	0.0008
rps8	57	400	390	13	0.0333	14	0.8208	0.0010	0.0037
rps8-rpl14	54	316	167	15	0.0898	14	0.6792	0.0047	0.0068

rrn16	51	1491	1491	2	0.0013	2	0.0392	0.0014	0.0001
rrn16-rrn23	32	2429	2414	10	0.0041	5	0.3407	0.0111	0.0003
rrn16-trnI^{gau}	19	296	296	1	0.0034	2	0.1988	0.0126	0.0007
rrn23	51	2809	2807	2	0.0007	3	0.0776	0.0026	0.0000
rrn4-5-rrn5	51	253	222	2	0.0090	3	0.1498	0.0043	0.0007
rrn5-trnR^{acg}	19	261	252	3	0.0119	4	0.5088	0.0137	0.0025
rrn5-ycf1	32	1390	1272	13	0.0102	11	0.7036	0.0068	0.0011
trnA^{ugc}	24	881	871	0	0.0000	1	0.0000	0.0000	0.0000
trnC^{gca}-petN	25	1052	811	50	0.0617	25	1.0000	0.0001	0.0080
trnD^{guc}-trnY^{gua}	25	469	425	29	0.0682	22	0.9800	0.0005	0.0063
trnE^{uuc}-trnT^{ggu}	24	687	491	22	0.0448	18	0.9239	0.0024	0.0039
trnF^{gaa}-ndhJ	25	755	632	51	0.0807	22	0.9800	0.0005	0.0084
trnG^{gcc}	18	786	753	21	0.0279	14	0.9673	0.0009	0.0041
trnG^{gcc}-trnR^{ucu}	20	359	179	25	0.1397	16	0.9474	0.0019	0.0215
trnH^{gug}-psbA	24	337	195	20	0.1026	17	0.9493	0.0010	0.0162
trnI^{gau}	24	1020	1020	6	0.0059	3	0.1630	0.0098	0.0005
trnK^{uuu}	24	2663	2540	107	0.0421	24	1.0000	0.0001	0.0058
trnK^{uuu}-matK	25	1530	1515	74	0.0488	25	1.0000	0.0001	0.0061
trnL^{caa}-ndhB	25	652	569	2	0.0035	3	0.1567	0.0092	0.0003
trnL^{uaa}	24	643	595	21	0.0353	17	0.9529	0.0010	0.0042
trnL^{uaa}-trnF^{gaa}	25	412	388	25	0.0644	18	0.9567	0.0008	0.0068
trnM^{cau}-atpE	25	263	204	6	0.0294	9	0.7067	0.0077	0.0055
trnN^{guu}-ycf1	19	327	321	0	0.0000	1	0.0000	0.0000	0.0000
trnP^{ugg}-psaJ	24	446	402	31	0.0771	19	0.9601	0.0010	0.0082

trnQ^{uug}-psbK	25	504	383	15	0.0392	13	0.7400	0.0095	0.0037
trnR^{acg}-trnN^{guu}	25	637	577	3	0.0052	2	0.0800	0.0052	0.0004
trnR^{ucu}-atpA	23	1187	271	30	0.1107	22	0.9960	0.0002	0.0170
trnS^{gcu}-trnG^{gcc}	20	706	584	30	0.0514	19	0.9947	0.0003	0.0083
trnS^{gga}-rps4	24	304	283	17	0.0601	15	0.8913	0.0033	0.0061
trnS^{uga}-psbZ	25	371	291	18	0.0619	14	0.8433	0.0050	0.0069
trnT^{ggu}-psbD	24	1842	1209	79	0.0653	24	1.0000	0.0001	0.0081
trnT^{ugu}-trnL^{uaa}	25	1671	756	83	0.1098	25	1.0000	0.0001	0.0128
trnV^{gac}-rrn16	19	227	227	0	0.0000	1	0.0000	0.0000	0.0000
trnV^{uac}	24	671	661	20	0.0303	17	0.9384	0.0015	0.0031
trnW^{cca}-trnP^{ugg}	25	250	140	21	0.1500	15	0.8467	0.0052	0.0156
ycf1	14	5646	5597	130	0.0232	14	1.0000	0.0007	0.0052
ycf2	57	6858	6831	31	0.0045	24	0.8690	0.0017	0.0003
ycf2-ndhB	33	1669	1612	10	0.0062	10	0.5189	0.0113	0.0004
ycf2-trnL^{caa}	24	951	946	7	0.0074	7	0.4457	0.0159	0.0006
ycf3	57	1996	1915	49	0.0256	32	0.9411	0.0005	0.0018
ycf3-rps4	31	1098	931	48	0.0516	25	0.9742	0.0004	0.0048
ycf3-trnS^{gga}	24	677	594	20	0.0337	14	0.8333	0.0059	0.0035
ycf4	58	555	555	15	0.0270	16	0.7731	0.0031	0.0023
ycf4-cemA	55	587	495	49	0.0990	38	0.9832	0.0001	0.0080
